# The Potential of Alkyl Amides as Novel Biomarkers and Their Application to Paleocultural Deposits in China

**DOI:** 10.1038/s41598-017-15371-z

**Published:** 2017-11-07

**Authors:** Jianjun Wang, Bernd R. T. Simoneit, Guoying Sheng, Liqi Chen, Libin Xu, Xinming Wang, Yuhong Wang, Liguang Sun

**Affiliations:** 1 Key Laboratory of Global Change and Marine-Atmospheric Chemistry, Third Institute of Oceanography, State Oceanic Administration, Xiamen, 361005 Fujian, China; 2 Institute of Polar Environment, University of Science and Technology of China, Hefei, 230026 Anhui China; 3 Department of Chemistry, Oregon State University, Corvallis, OR 97331 USA; 4 Guangzhou Institute of Geochemistry, Chinese Academy of Sciences, Guangzhou, 510640 Guangdong, China; 5 NIH Chemical Genomics Center, National Institute of Health, Bethesda, MD 20892 USA

## Abstract

A series of alkyl amides was detected and identified in the sedimentary record from an archaeological site at Yuchisi, Mengcheng, Anhui, China. The alkyl amides profiles change abruptly at the depth corresponding to the transition between two prehistoric cultures, which also corresponds to an abrupt change in the fatty acid ratio C_18:2_/C_18:0_. The different patterns of variation of the longer and shorter chain alkyl amides at the depth of the cultural transition may reflect differences in their response to external environmental changes, as well as different sources. This is the first study of the stratigraphic variation of alkyl amides in sediments, and their first application to assess paleoenvironmental changes. We suggest that alkyl amides may have potential as new biomarkers in archeological and paleoenvironmental studies.

## Introduction

In organic environmental chemistry, most of the commonly-used molecular biomarkers, such as hydrocarbons, alcohols and acids, are composed of C, H and O^1^. Although nitrogen is also one of the most important and abundant elements in living organisms, nitrogen-containing biomarkers or indicators are rarely studied^1^. In sediments, ammonium or amino acids are common forms of nitrogen-containing organic material. They are readily degraded and transformed and therefore are not useful as environmental indicators.

Recently, a series of nitrogen-containing organic compounds, alkyl amides, were detected in environmental samples and were proposed as potential environmental indicators. Abas and Simoneit reported the occurrence of alkyl amides in haze aerosols caused by tropical fires and proposed them as novel tracers of biomass burning^2,3^. Biogenic alkyl amides, with carbon numbers ranging from 12 to 22:1 (C_22:1_ dominant), were reported in ambient urban aerosols in Santiago, Chile, and were attributed to cooking/grilling emissions^3,4^. Source tests of emissions from grilling/cooking of meat, but not deep-frying with vegetable oils, also contained alkyl amides^5–7^. Buckley *et al*. reported that alkyl amides (C_16_ dominant to C_18_) were volatilized from organic matter from Egyptian Pharaonic mummies^8^, and Makou *et al*. detected the same compounds in alpine ice cores^9^. Generally, alkyl amides are proposed to originate from reactions between fatty acids and ammonia occurring naturally in biomass burning and the subsequent fallout of particles to soil surfaces, ice fields, or stream water^3,9–13^. However, alkyl amides in sediments are not well studied and have not been previously regarded as a potential environmental tracer for paleoenvironmental and paleoclimatic studies^3,10,12,14^. Considering the stabilities of alkyl amides and their occurrence in aerosols and sediments, we proposed that they could have potential as indicators of paleoclimate and paleoenvironment in sedimentary deposits. Here, we test this hypothesis at Yuchisi, a well-studied Neolithic archaeological site in central China.

**Figure 1:**
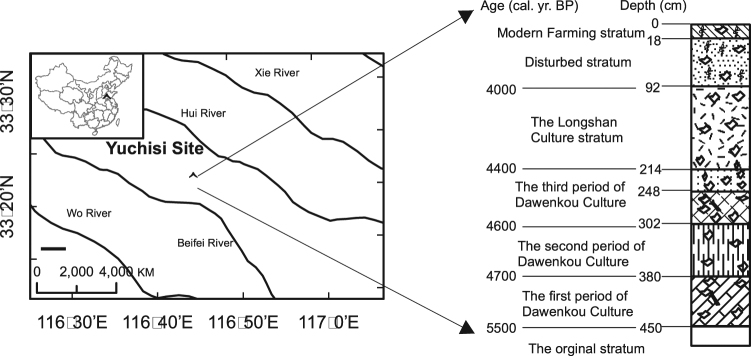
Location of Yuchisi archeological site and profile YC1. Right: Stratigraphy, chronology, and cultural context of the studied sedimentary deposit (the stripes of the sediment profile were slightly modified and the “layers” in the image has been changed to “stratum”). The map was created with ArcGIS 10.3.1 (http://www.esri.com/software/arcgis). (Reused from refs^15,16^ under reprinted under a Creative Commons Attribution 4.0 International License https://creativecommons.org/licenses/by/4.0/).

The Yuchisi archeological site is in Mengcheng County (32 °55′−33 °29′N, 116 °16′−116 °46′E), Anhui Province, China (Fig. 1). It is located between the Yellow and Huai Rivers and has an area of ~100,000 m^2^; it is the largest, most integrated site with abundant prehistoric architectural remains in central China. Since 1990, archeologists have explored the site 13 times and discovered numerous porcelain shards and bones. Based on archeological evidence, the Yuchisi site was proposed as an important geographical location, a proto-city, which was a focus of human settlement during the Neolithic period. Most importantly, Yuchisi was a regional cultural center and provides a record of two of the important prehistoric cultures in China: the Late Dawenkou Culture (5,050–4,400 yr B.P.) and the Longshan Culture (4,400–4,000 yr B.P.). Previous studies demonstrated that environmental and climate changes may have contributed to the transition from the Dawenkou Culture to the Longshan Culture^15–17^. Therefore, Yuchisi is an excellent site for testing the possible use of alkyl amides as indicators of paleoenvironmental and paleoclimatic changes.

In this study, we sampled a 460-cm-long sediment profile, YC1 (Fig. 1). Using gas chromatography-mass spectrometry (GC-MS), we report on the detection and identification of alkyl amides as trimethylsilyl (TMS)-derivatives and present the results as depth profiles. We also discuss the occurrence of discrepancies between the profiles of the short and long chain amides, their possible sources, and the potential of alkyl amides as markers for archeological and paleoenvironmental studies.

## Results

### Identification of alkyl amides in sediment

**Figure 2:**
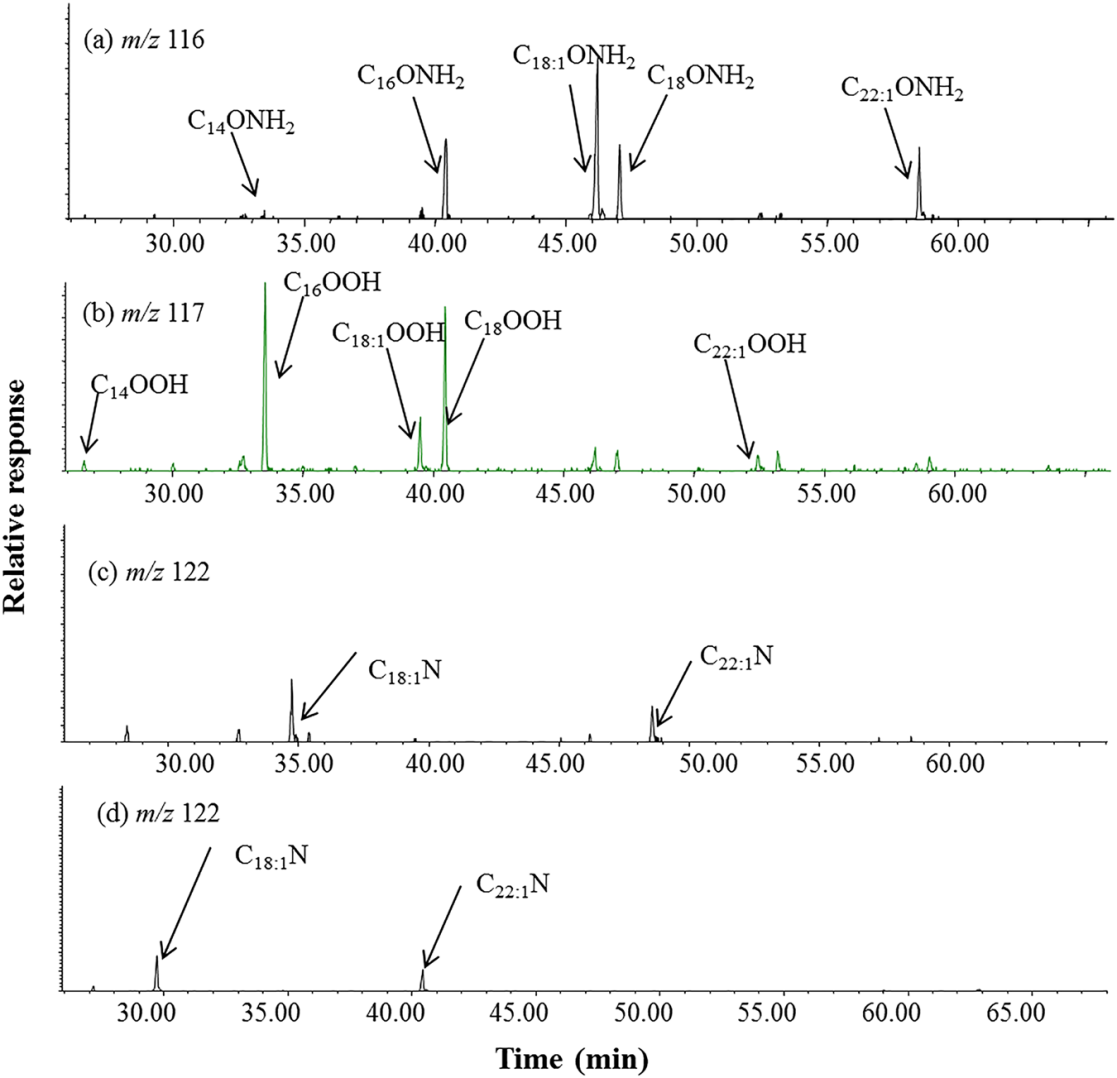
Distributions of lipid tracers in profile YC1: (**a**) alkyl amides in sample YC1-225 (m/*z* 116, as TMS); (**b**) fatty acids in sample YC1-225 (*m/z* 117, as TMS); (**c**) alkyl nitriles in sample YC1-225 (*m/z* 122, in the acid fraction); (**d**) alkyl nitriles in sample YC1-225 (*m/z* 122, in the alkanol fraction).

**Figure 3:**
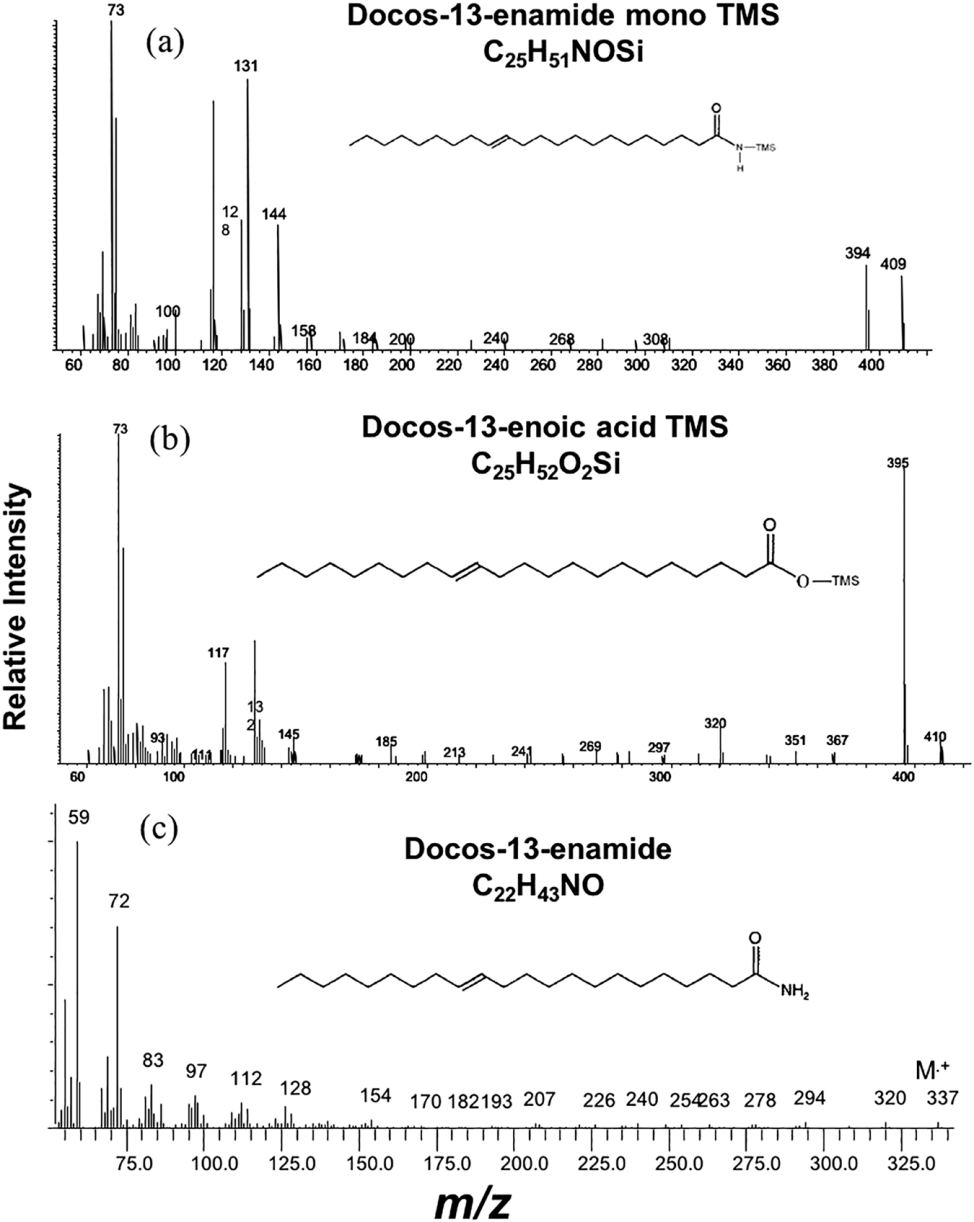
Mass spectra of (**a**) docos-13-enamide-TMS derivative (standard), (**b**) docos-13-enoic acid-TMS ester, and (**c**) docos-13-enamide^3^.

The sediment samples from Yuchisi were freeze-dried, Soxhlet extracted, saponified, separated into alkane, alcohol and acid fractions, and then analyzed by GC-MS. The acid fraction of the samples had significant peaks with intense fragment ions at *m/z* 73, 116, 131, and 144, besides the normal TMS esters of fatty acids (key ion at *m/z* 117) (Fig. 2a,b). The mass spectra of these peaks have a fragmentation pattern similar to those of fatty acid TMS esters, but a molecular weight 1 Da less (Fig. 3a,b). Interpretation of this fragmentation pattern suggests that these peaks are alkyl amides.

The molecular weight of erucamide is 337, with key ions at *m/z* 59 and 72 in its mass spectrum^3^. After silylation, the characteristic mass spectra of oleamide and erucamide had ions at *m/z* 73, 116, 131, and 144, and M^.+^ at *m/z* 353 and 409, respectively (Fig. 3 and Supplemental Material, Fig. SM-1), corresponding to those of the peak series in the YC1 samples. Based on these data, we chose the *m/z* 116 key ion as characteristic of alkyl amide mono-TMS derivatives (e.g. Fig. 2a). The retention times of the alkyl amides-TMS with two less carbons are very close to those of the fatty acids-TMS (e.g. the retention time of C_16_ amide is 40.33 min while the retention time of C_18_ acid is 40.40 min) (Fig. 2a vs. b).

Alkyl nitriles were detected in the total extract, alcohol and acid fractions of the samples with the alkyl amides (Table 1). The series mainly comprises two compounds, oleonitrile (C_18:1_) and erucanitrile (C_22:1_) (e.g., key ion *m/z* 122, Fig. 2c,d and SM-2), with the same carbon number maxima as the parent alkyl amides. Alkyl nitriles were proposed to form by dehydration of alkyl amides during pyrolysis^5–7^. The occurrence of alkyl nitriles in environmental samples has been reported and their origin attributed to the thermal alteration (e.g., by burning) of organic matter containing alkyl amides^3^. In the present study, based on a doping test with standards, the similar distributions of both nitriles and amides indicated that the nitriles formed from the dehydration of the corresponding alkyl amides. Therefore, the amounts of alkyl amides and corresponding nitriles were summed to represent total alkyl amides.

**Table 1:** Concentrations of total alkyl amides, fatty acids and alkyl nitriles in the sediments of profile YC1.

Sample	Depth (cm)	Alkyl amides (μg/g)	Fatty acids (μg/g)	Alkyl nitriles (μg/g)
YC1–5	8–10	2.57	8.32	0.82
YC1–20	38–40	4.18	7.69	0.74
YC1–30	58–60	5.42	5.21	1.65
YC1–45	88–90	4.78	5.02	1.36
YC1–60	118–120	4.11	6.03	1.18
YC1–75	148–150	4.84	6.60	1.13
YC1–100	198–200	9.17	14.94	2.58
YC1–105	208–210	7.43	10.54	1.62
YC1–107	212–214	4.28	4.63	2.08
YC1–110	218–220	5.52	4.79	1.58
YC1–135	268–270	3.84	6.80	1.28
YC1–150	298–300	1.98	6.21	0.67
YC1–165	328–330	8.16	12.41	2.17
YC1–180	358–360	2.63	5.72	0.76
YC1–225	448–450	6.54	8.20	1.98
YC1–230	458–460	4.20	7.14	1.74

### Composition and distribution patterns of alkyl amides and fatty acids in profile YC1

The composition and distribution patterns of alkyl amides and fatty acids are overall comparable in the sediments of YC1, and their concentrations are similar. Both series have even-to-odd carbon number preferences and range from C_14_ to C_22_ (Fig. 2). The alkyl amides have the major homologs of C_16_, C_18:1_, C_18_ and C_22:1_, and the fatty acids are mainly C_16_ and C_18_ (Fig. 2). The unsaturated C_18_ amides consist of three peaks (Fig. 2), the same as the unsaturated C_18_ acids. In addition, the C_22:1_ amide and C_22:1_ acid have the double bond position at C-13 for both. This suggests a precursor-product relationship among the C_18:1_ and C_22:1_ amides and acids, respectively. The concentrations of C_14_ and C_20_ alkyl amides were too low for detection. The homolog distributions of the alkyl amides, as in the case of the fatty acids, indicate a biogenic origin.

**Figure 4:**
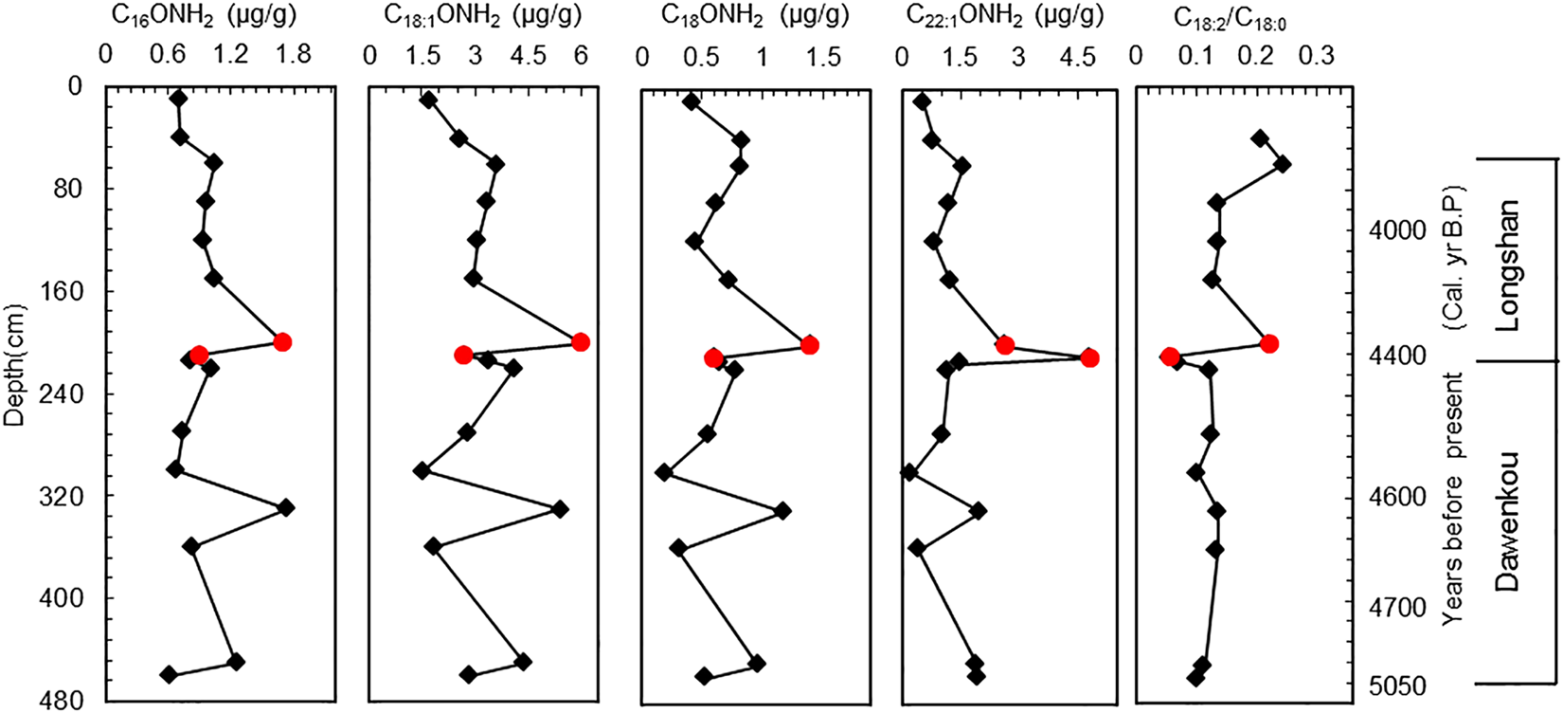
Concentration profiles of the alkyl amides (the sum of the alkyl amides and nitriles) (μg/g) and the fatty acid ratio for C_18:2_/C_18:0_ in the Yuchisi sediments (YC1).

In profile YC1, unsaturated C_18:1_ amides are the dominant ones, followed by unsaturated C_22:1_ amide. The concentrations of the C_18:1_ and C_22:1_ amide range from 1.53 to 6.01 μg/g and 0.25 to 4.83 μg/g, respectively. The saturated C_16_ and C_18_ amides are less abundant with concentrations ranging from 0.61 to 1.73 μg/g and from 0.20 to 1.39 μg/g, respectively. The three shorter chain (C_16_, C_18:1_ and C_18_) alkyl amides exhibit generally similar concentration-versus-depth profiles, and three peaks are evident, at depths of 218–220 cm, 328–330 cm and 448–450 cm. Longer chain C_22:1_ amides exhibit a similar profile as the three shorter chain forms, except for a clear difference at the depths of 198–210 cm. The concentrations of the C_16_, C_18_ and C_18:1_ alkyl amides increase from 208–210 cm to 198–200 cm, while the C22:1 amide decreases from 208–210 cm to 198–200 cm (Fig. 4).

## Discussion

### Alkyl amide profiles and the paleoclimate of Yuchisi

During several decades around 4,400 yr BP, remarkable climatic changes occurred worldwide, including drought, floods, dust storms, and possibly other climatically-related anomalies. Many major prehistoric cultures such as the Akkadian empire in Mesopotamia, the pyramid-constructing Old Kingdom civilization of Egypt, the Harappan 3B civilization of the Indus valley, and the Early Bronze III civilizations of Palestine, Greece, and Crete, reached peaks in development around 4,300 yr BP, but then terminated abruptly before 4,200 yr BP, as a result of catastrophic drought and cooling^15,18–20^. The archaeological evidence indicates that the sedimentary sequence at Yuchisi records two important Chinese Neolithic cultures: the interval of 450–214 cm corresponds to the late Dawenkou Culture (5,050–4,400 yr B.P.), and the interval of 214–92 cm to the Longshan Culture (4,400–4,000 yr B.P.). Previous studies showed that biomarkers of the fatty acid ratio C_18:2_/C_18:0_, alkane ratio 2C_31_/(C_27_ + C_29_), *n*-C_18_-ol and *n*-C_30_-ol, as well as pollen assemblages, exhibited dramatic changes at the depths of the transition between the two cultures (about 4,400 yr B.P.). This suggests that the cultural changes recorded in different regions may have been synchronous and were a response to climatic and environmental changes^15^. Our previous investigation also suggested that dramatic changes in climate and environment within a short interval also contributed to the transition between the Dawenkou Culture and Longshan Culture at Yuchisi site. In addition, the results of the present study suggest that the observed changes in alkyl amides within profile YC1 were responses to climatic and cultural changes during this interval.

Erucamide was the dominant amide in some urban aerosols and was proposed as a tracer for emissions from biomass burning or cooking (erucic acid is, for example, the main acid in rapeseed oil) and hence the C_22:1_ amide in aerosols^7^; in addition, shorter chain amides (C_16_, C_18:1_ and C_18_) dominate the amide composition of mummies^8^ and penguin droppings (Fig. SM-3). The short chain amides apparently are more strongly correlated with animal sources such as humans and seabirds, and the additional longer chain (erucic) amides are from vegetation sources associated with food preparation or the burning of plant biomass (possibly for heating in cold weather). Archeological evidence at Yuchisi site shows that agriculture was well developed during the Longshan and Dawenkou cultures. Black charcoal was found in many layers in the Yuchisi sediment profile, in addition to carbonized seeds^21^. The different sources of the short and long chain alkyl amides may reflect their different responses to paleoclimatic or anthropogenic changes (the ratio of the short versus long chain alkyl amides may also reflect different aspects of paleoclimatic or anthropogenic change), leading to different concentration profiles in the record of YC1.

### Potential of alkyl amides and nitriles as environmental tracers

Earlier reports documented the formation of alkyl amides under hydrothermal conditions^14^ and in aerosols from biomass burning^3^, implicating higher temperature processes, or via amidation of esters with sedimentary ammonia derived from anaerobic degradation of organic matter in thermally-unaltered sediments^12^. Simoneit *et al*.^3^ treated fatty acids plus aqueous (NH_4_)HCO_3_ in confined vessels at 300 °C for 3 days and detected alkyl amides and nitriles. They proposed that amides and nitriles are formed in the reactions of fatty acids with ammonia or ammonium salts (possibly from protein decomposition) during burning and combustion processes. These reactions might also occur during early diagenesis of organic detritus in soils and sediments. The similar distribution patterns of alkyl amides and alkanoic acids in our samples support this hypothesis. High levels of nitrogen in human feces and domestic animal dung at the site may have provided sufficient ammonia or ammonium salts to react with alkanoic acids and form alkyl amides within the sediment profile.

However, the formation mechanism and sources of the alkyl amides in these sediments remain unclear without further research. The sparsity of reports of sedimentary alkyl amides might be due to two reasons. First, the concentrations of alkyl amides are usually lower than those of fatty acids (e.g., in fresh penguin droppings or related ornithogenic sediments - Wang *et al*., unpublished results). Second, C_n_ alkyl amides have a GC retention time quite close to that of C_n+2_ fatty acids, both as TMS derivatives. Hence, the effective separation of amides from fatty acids is needed for further study of alkyl amides.

An ideal tracer compound should be stable, have a simple source, and be environmentally significant. The presence of alkyl amides in the Yuchisi sediments dating to 5,050 yr B.P. indicates that they are stable over geological time. Compared to fatty acids which have complex sources, alkyl amides have relatively simple sources. For example, the sources of saturated fatty acids, especially shorter chains (C_16_ and C_18_), may be plants, algae, or even bacteria. The chemical composition and possible formation mechanisms suggest that alkyl amides are directly or indirectly related to higher temperature processes and/or protein-derived nitrogen such as ammonia or ammonium. In our sampling environment, this suggest a possible connection with human activities such as cooking and the presence of human/animal feces. Furthermore, in living organisms, unsaturated acids (e.g., C_18:1_) usually exhibit a higher concentration than the corresponding saturated acid (i.e., C_18:0_) and readily form amides. Therefore, alkyl amides may be more sensitive indicators of the dynamics of living organisms and potentially broaden the application of alkyl amides in other environments.

To the best of our knowledge, this is the first study of alkyl amides as TMS-derivatives in extracts of sediment samples which assesses their temporal distribution. The depth profiles of the alkyl amides in the Yuchisi sediments show that they are sensitive to paleoclimatic changes and thus have potential as tracers for paleoenvironmental studies. However, it is noteworthy that although their concentrations are comparable in the Yuchisi sediments, the concentrations of alkyl amides are much lower than those of fatty acids in other sediment samples investigated, such as fresh penguin droppings or related ornithogenic sediments in the Antarctic (Fig. SM-3). In addition, our understanding of the sources and formation of alkyl amides in sediment samples are still limited. The variations of alkyl amides concentrations in the Yuchisi sediments need further investigation to determine their precise paleoclimatic and paleoenvironmental significance. In addition, more investigations of sediment samples from different environmental settings and sedimentation conditions are required to fully evaluate the application of alkyl amides as environmental tracers.

## Methods

### Sampling site

The sampling site at Yuchisi is located roughly at the center of a hill, and is 2–3 m above ground level. The hill has dimensions of ~370 m (east-west) and ~250 m (north-south). The site is bounded on its western and southern sides by minor unsurfaced roads, and on its eastern side by a modern artificial canal (Fig. 1). The remains of an ancient moat were found around the site. The moat extends 230~240 m north-south, 220 m east-west, and has a width of 25–30 m, a depth of 4.5 m, and forms a circle with an outlet in the south-west corner; the architectural complex of the late Dawenkou Culture is surrounded by this moat. Along the north-south axis and in the center of the moat are three archeological excavation pits. Sediment profile (YC1) is from the south wall of the central excavation pit. It is 4.6 m deep, and its cultural layers and ages have been studied in detail. From top to bottom, the pit was divided into 8 layers or strata (Fig. 1), and the boundaries of each stratum were assigned in the field under the direction of Prof. Wang Jihuai from the Archaeological Research Institute, Chinese Academy of Social Science, Beijing, China^20^. 230 samples were obtained from the profile, at an interval of 2 cm. In this study, we analyzed 16 samples from the profile; the sampling depths were selected based upon previous TOC analysis and archeological exploration^16^.

### Sample preparation and analysis

Prior to analysis, the sediment samples were stored in precleaned brown glass jars. For organic analysis, freeze-dried sediment samples were Soxhlet extracted for 72 h with dichloromethane/methanol (DCM/MeOH, 2:1 vv). The extracts were concentrated by rotary evaporation and then saponified using 0.5 M KOH/MeOH. Neutral lipids were partitioned out of the basic solution with hexane. The pH of the saponified extract was then brought to 2 by 6 N HCl and acidic lipids were extracted with 20% DCM in hexane. The acidic lipids, which contain the amides, were treated overnight with anhydrous Na_2_SO_4_ to remove water. Neutral lipids were further separated by (5% deactivated) silica gel column chromatography using solvents of increasing polarity from hexane to DCM. The acid (with amides) and alcohol fractions were treated with *N*,*O*-bistrimethylsilyltrifluoroacetamide (BSTFA) to form trimethylsilyl (TMS)-ester and ether derivatives, respectively, prior to gas chromatography-mass spectrometry (GC-MS) analysis.

For identification of alkyl amides, oleamide (*cis-*9-octadecenamide, CAS: 301-02-0), erucamide (docos-13-enamide, CAS: 112-84-5) were purchased from Sigma-Aldrich. The standards of oleamide and erucamide were subjected to the experimental procedure and also doped on an extracted sediment sample for recovery tests. The standard test samples were Soxhlet extracted, saponified, and separated by silica gel column chromatography. Then, the samples were treated with BSTFA to form TMS derivatives and analyzed by GC-MS.

The recovery tests of oleamide and erucamide from the extracted sediment sample using the same extraction and separation scheme were semi-quantitative (75–88%). No alkyl amides were detected in the procedural blank sample. However, alkyl nitriles were generated (up to 30%) during the analytical procedure from the standards and natural amides in the total extract, alcohol and acid fractions. The mass spectra of alkyl nitriles have odd molecular ions, successive even C_n_H_2n_ fragments, and key ions at *m/z* 70, 110 for saturated and *m/z* 69, 122 for unsaturated homologues, respectively. We used *m/z* 122 as the key ion for GC-MS detection of nitriles. The GC-MS data for the oleamide and erucamide standards contained the corresponding alkyl nitriles, generated during analysis from the amides. Thus, the concentrations of the total alkyl amides were calculated as the sum of the alkyl nitriles plus amides.

The silylated extract fractions were analyzed on an HP 5890 GC-MS system with a DB-5MS (50 m × 0.32 mm and 0.25 μm film thickness) capillary column (J&W). Helium was used as the carrier gas. The MS was operated in electron impact mode at 70 eV ionization energy. The GC oven was programmed as follows: hold 2 min at 60 °C, increase to 150 °C at 10 °C/min, increase to 300 °C at 2.5 °C/min, hold 30 min at 300 °C. The MS data was acquired in the full scan mode and processed with the Chemstation data system.

## Electronic supplementary material

Supplemental information
