# Supplementary material for: The Potential of Alkyl Amides as Novel Biomarkers and Their Application to Paleocultural Deposits in China
Source: Sci Rep. 2017 Nov 7;7:14667. doi: 10.1038/s41598-017-15371-z (PMC5677102; doi:10.1038/s41598-017-15371-z)

Supplemental Material for

The Potential of Alkyl Amides as Novel Biomarkers and Their Application to Paleocultural Deposits in China

Jianjun Wang[1,2*], Bernd R.T. Simoneit[3], Guoying Sheng[4], Liqi Chen[1], Libin Xu[2], Xinming Wang[4], Yuhong Wang[5], Liguang Sun[2*]

[1] Key Laboratory of Global Change and Marine-Atmospheric Chemistry, Third Institute of Oceanography, State Oceanic Administration, Xiamen 361005, Fujian, China

[2] Institute of Polar Environment, University of Science and Technology of China, Hefei 230026, Anhui, China

[3] Department of Chemistry, Oregon State University, Corvallis, OR 97331, USA

[4] Guangzhou Institute of Geochemistry, Chinese Academy of Sciences, Guangzhou 510640, Guangdong, China

[5] NIH Chemical Genomics Center, National Institute of Health, Bethesda, MD 20892, USA

* Correspondence and requests for materials should be addressed to J.W. (email: wangjianjun@tio.org.cn) or L.S. (email: slg@ustc.edu.cn)

Contents: 3 Figures

Figure SM-1. Characteristic mass spectra of alkyl amides as TMS in YC1

(a) Palmitamide ($C_{19}H_{41}NOSi$)—$C_{16}ONH_2$

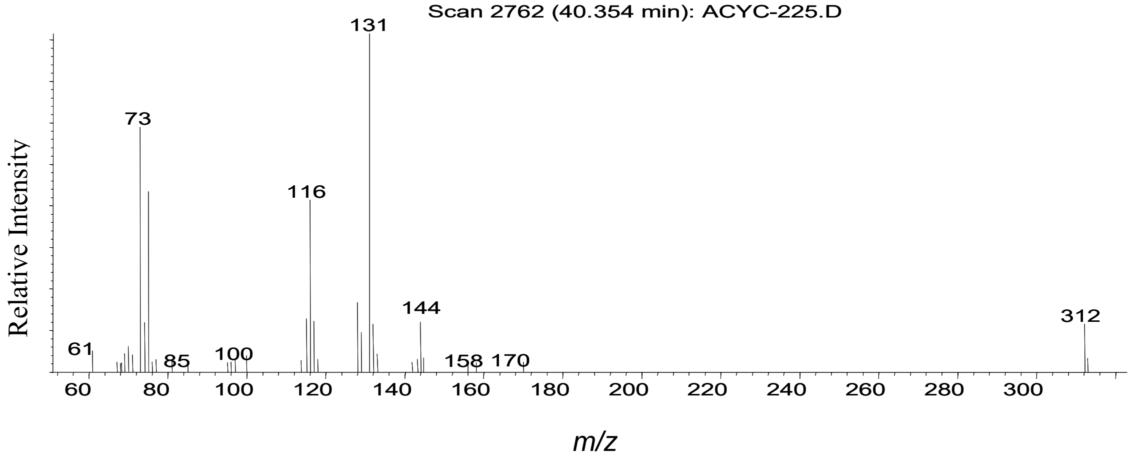

(b) Linoleamide ($C_{21}H_{41}NOSi$)—$C_{18:2}ONH_2$

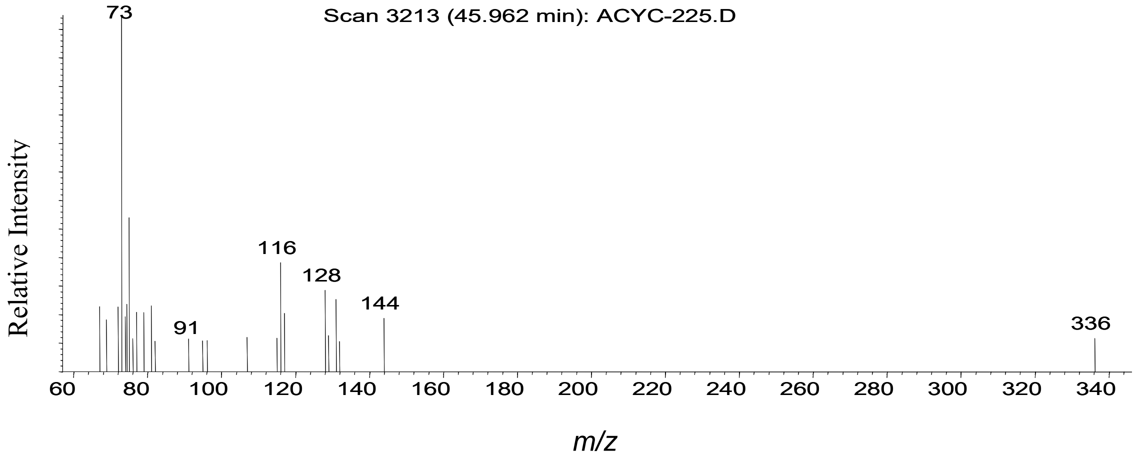

(c) Oleamide ($C_{21}H_{43}NOSi$)—$C_{18:1}ONH_2$

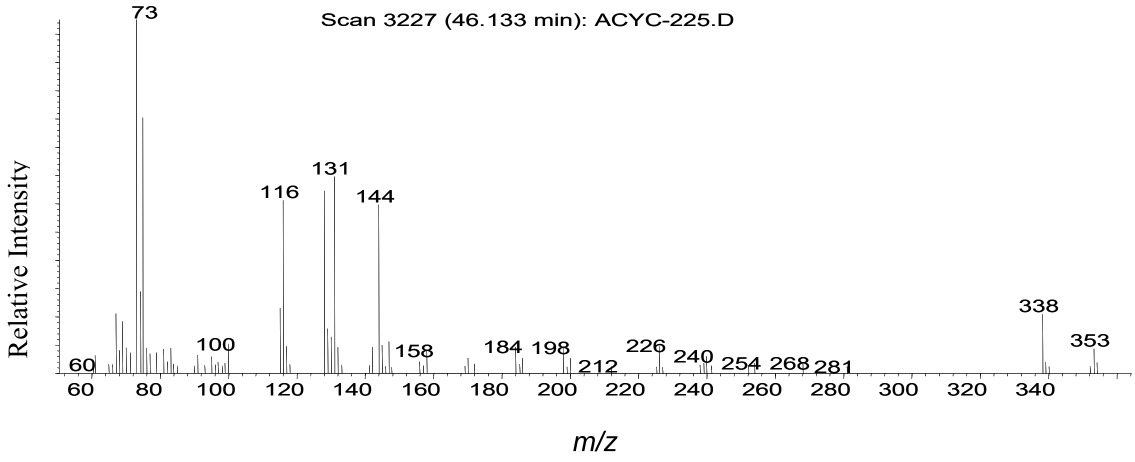

(d) Stearamide ($C_{21}H_{45}NOSi$)—$C_{18}ONH_2$ (Octadecanamide)

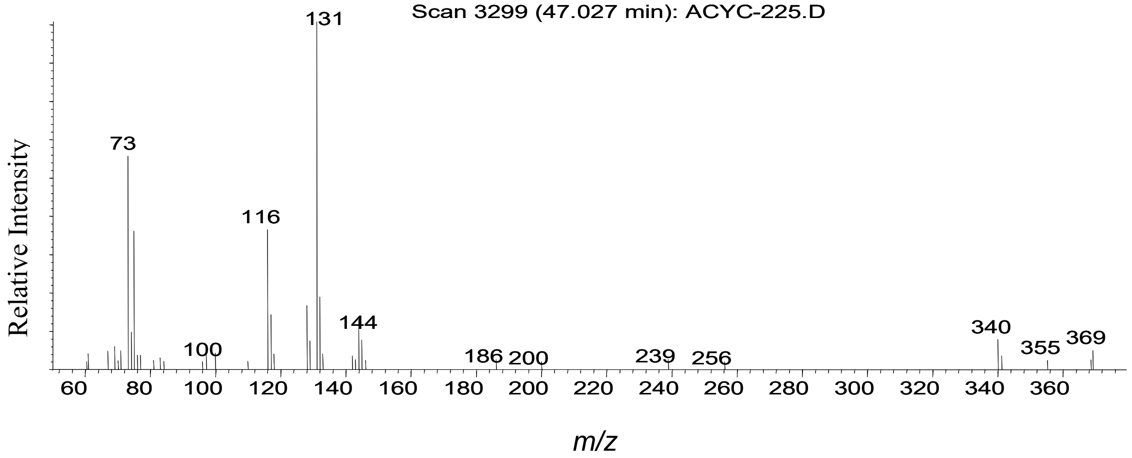

(e) Erucamide ($C_{25}H_{51}NOSi$)—$C_{22:1}ONH_2$

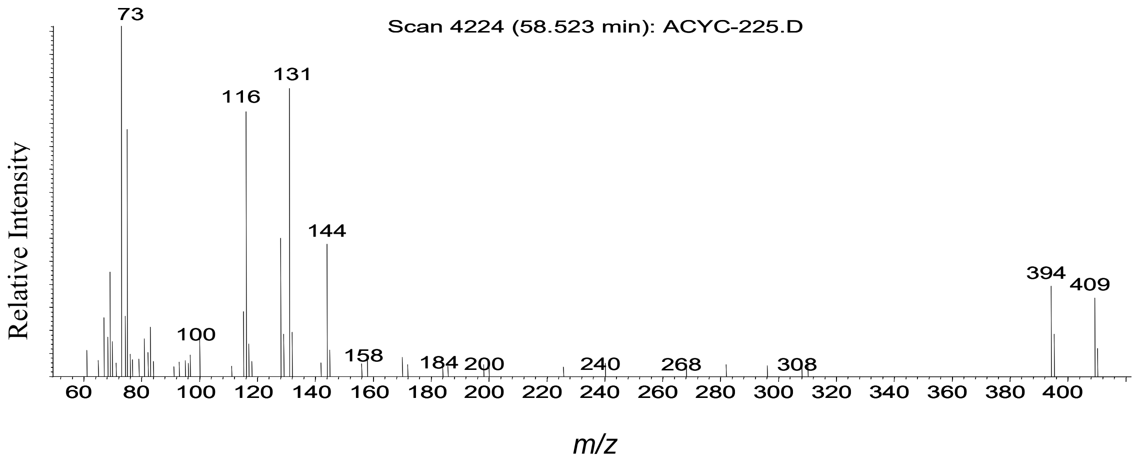

Fig. SM-2. Alkyl nitriles and alkanols as TMS in YC1

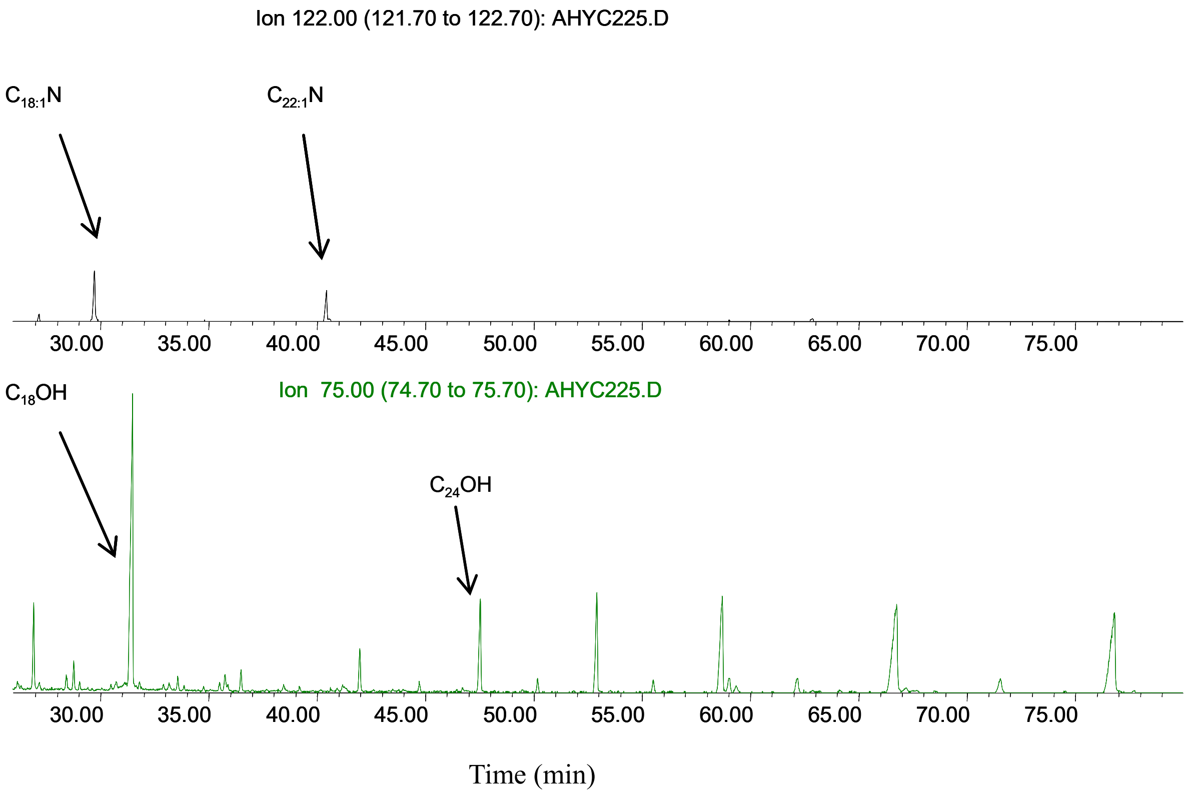

Note -$C_{18:1}$ = oleonitrile, $C_{22:1}$ = erucanitrile.

Figure SM-3: Distributions of amides and acids in penguin droppings: (a) alkyl amides (m/z 116, as TMS); (b) fatty acids (m/z 117, as TMS)

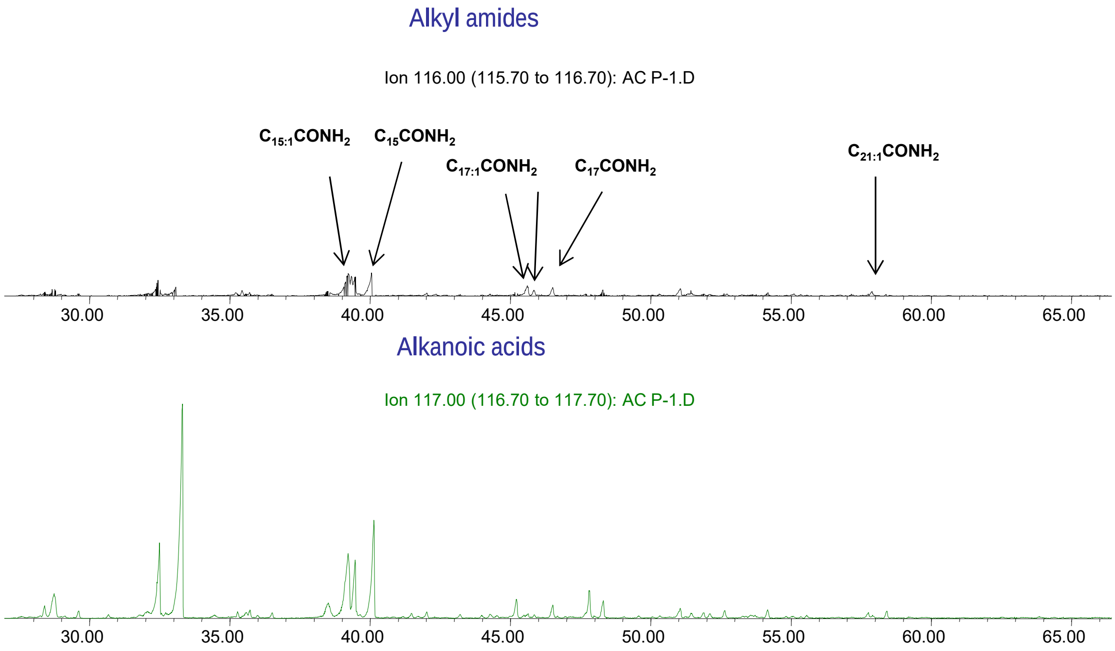

Supplement: Supplementary file 1 — Supplemental information [file 41598_2017_15371_MOESM1_ESM.pdf]
